# Coral responses to a catastrophic marine heatwave are decoupled from changes in total coral cover at a continental scale

**DOI:** 10.1098/rspb.2024.1538

**Published:** 2024-10-09

**Authors:** Camille Mellin, Rick D. Stuart-Smith, Freddie Heather, Elizabeth Oh, Emre Turak, Graham J. Edgar

**Affiliations:** ^1^ The Environment Institute and School of Biological Sciences, University of Adelaide, Adelaide, South Australia 5005, Australia; ^2^ Institute for Marine and Antarctic Studies, University of Tasmania, Hobart, Tasmania, Australia

**Keywords:** beta diversity, community ecology, coral bleaching, marine heatwave, ocean warming, species turnover

## Abstract

The services provided by the world’s coral reefs are threatened by increasingly frequent and severe marine heatwaves. Heatwave-induced degradation of reefs has often been inferred from the extent of the decline in total coral cover, which overlooks extreme variation among coral taxa in their susceptibility and responses to thermal stress. Here, we provide a continental-scale assessment of coral cover changes at 262 shallow tropical reef sites around Australia, using ecological survey data on 404 coral taxa before and after the 2016 mass bleaching event. A strong spatial structure in coral community composition along large-scale environmental gradients largely dictated how coral communities responded to heat stress. While heat stress variables were the best predictors of change in total coral cover, the pre-heatwave community composition best predicted the temporal beta-diversity index (an indicator of change in community composition over time). Indicator taxa in each coral community differed before and after the heatwave, highlighting potential winners and losers of climate-driven coral bleaching. Our results demonstrate how assessment of change in total cover alone may conceal very different responses in community structure, some of which showed strong regional consistency, and may provide a telling outlook of how coral reefs may reorganize in a warmer future.

## 1. Introduction

Climate-driven coral bleaching is now regarded as the number one threat to the world’s coral reefs, being the most prominent agent of the decline in coral cover [1,2] and associated coral reef biodiversity [3–5]. Marine heatwaves that cause elevated ocean temperatures of only 1–2°C above long-term maxima can induce the paling of scleractinian corals, which results from the breakdown of their symbiosis with dinoflagellate microalgae, and can lead to coral mortality if thermal stress is prolonged [6]. The global rise in marine heatwave frequency and severity has led to an eightfold increase in the area of coral reefs likely exposed to mass bleaching events since the 1998 El Niño event, compared to the 1985–1997 period [7]. Since coral reefs host thousands of fish and invertebrate species and provide critical ecosystem services (e.g. fisheries, tourism and physical protection against tropical storms and cyclones [8]), substantial concerns have been raised by the scientific community regarding the viability of coral reef ecosystems in a warming ocean [1,9,10].

While regional declines in total hard coral cover have been widely documented as a conspicuous response to climate-driven coral bleaching, they fail to reveal among-species variation in coral susceptibility to heat stress (e.g. [11,12]). Hard coral cover loss is strongly linked to the accumulated thermal stress experienced during a marine heatwave, which is commonly quantified through degree heating weeks (DHW) [13]. Greater DHW generally results in a greater proportion of bleached (and potentially dead) coral colonies; however, this relationship exhibits considerable taxonomic, spatial and temporal variation [14,15]. One potential cause for this variation is the footprint of past disturbances (‘ecological memory’ [16]), whereby less sensitive taxa that persist after previous disturbances exhibit a lower response to heat stress. In turn, the cumulative impacts of all previous disturbances (acute and chronic) that affected the coral community prior to the heatwave contribute to determining the proportion of its taxa with greater heat tolerance [11,12,17,18]. Determinants of heat tolerance in reef-building corals are indeed complex and include life history traits (e.g. [19,20]), short-term acclimation, long-term genetic adaptation and/or variation in symbiont community structure [21]. Therefore, pending sufficient time following a disturbance, across-species variation in heat tolerance could lead to important shifts in coral community composition even in the absence of changes in total hard coral cover; however, such shifts have been rarely documented (see, e.g. [11,22]).

Previous investigations of the variability in coral responses to heat stress have most often been conducted at the growth form level (e.g. tabular, branching and massive) [22–24]. However, coarse-resolution groupings can be misleading due to the within-group variation in coral susceptibility to heat stress and vulnerability status [19,25]. Furthermore, the three-dimensional complexity of corals varies greatly even at the genus level, implying variation in their potential habitat value for reef-associated organisms such as fishes [26,27]. A better understanding of how different coral taxa respond to heat stress is needed, because this taxon-specific variation in susceptibility will likely shape the structure of future coral reef communities as environmental filtering increasingly selects for stress-tolerant and/or resilient taxa. Barriers that hinder our understanding include (i) a paucity of standardized coral community data at large spatial scales, (ii) potentially confounding effects of environmental gradients on the structure of coral communities, (iii) spatial variation in both coral community structure and the magnitude of heat stress, and (iv) challenges identifying coral taxa.

Here, we quantified the relative abundance and composition of 119 reef-building coral categories (i.e. genus and growth-form combinations) at a continental scale across Australia’s tropical reefs, before and after the 2016 marine heatwave that impacted reefs on the east and west coasts [2,28,29]. We analysed changes in community structure in response to disturbance, in addition to the more commonly considered trends in total live coral cover. Based on broad-scale environmental gradients, we identified distinct coral communities that showed very different responses to heat stress, which we show were largely driven by pre-heatwave community composition.

## 2. Material and methods

### (a) Survey design

Coral communities were surveyed before and after the 2016 marine heatwave at a total of 262 sites across Northern Australia, encompassing four main regions that include Ningaloo Reef, the Northwest Shelf, the Great Barrier Reef and the Coral Sea. Pre-heatwave data were obtained between October 2010 and December 2015, and post-heatwave data were obtained between June 2016 and August 2019. Sites where other disturbances occurred between the pre- and post-heatwave surveys (e.g. Tropical Cyclone Debbie in 2017 and Tropical Cyclone Owen in 2018) were excluded from the analysis.

The impacts of other disturbances between the pre- and post-heatwave surveys at these sites are likely to be negligible for the following reasons:

— **2017 marine heatwave:** Most 2017 surveys in the Coral Sea and Great Barrier Reef regions were conducted in January–February 2017, i.e. before the 2017 heatwave that peaked in mid-March [30]. The only sites surveyed after mid-March 2017 were in the Coral Sea (CS1−4 and CS6−8) and escaped heat stress (DHW < 2.3°C-week according to NOAA Coral Reef Watch).
— **Tropical cyclones:** Based on the cyclone history for the entire survey area in the Great Barrier Reef/Coral Sea regions over the study period, there were no large cyclones (>Cat 3) that would have affected our sites. Surveys were completed before Cyclone Debbie (2017), and the only surveys done before Yasi (2011) were in areas outside of the destructive path of this cyclone (Lizard Is, Port Douglas, Whitsundays and Keppel). Cyclone history is available from the Bureau of Meteorology website (http://www.bom.gov.au/cyclone/history/index.shtml). On the Northwest Shelf, the only sites with >25 h of damaging waves (>4 m) for the entire study period based on Puotinen *et al*. [31] were those surveyed in September and October 2013, i.e. >8 months after Tropical Cyclone Narelle (January 2013) and before the 2016 heatwave. Therefore, those damaging waves occurred prior to the pre-heatwave surveys, with no other noticeable cyclone damage between those and the post-heatwave surveys. A few sites (*n* = 3–5) in Coral Bay Ningaloo Marine Park were affected by Tropical Cyclone Olwyn in 2015, but given their low number, they are unlikely to drive any directional changes in our analysis.
— **Crown-of-thorns starfish (CoTS) outbreaks:** CoTS were found in extremely low abundance among the data used for the study (mean 1.4 individuals per 50 m^2^ when present, only at 15 sites [3]). While it is possible that CoTS could have come through and reduced live coral cover at one or two sites between our surveys (without large numbers being detected before or after), this would be extremely unlikely to have any detectable impact on our findings if only affecting a small number of transects.

### (b) Data acquisition

All surveys were conducted using the standardized underwater visual census methods applied globally by Reef Life Survey, as previously described [32]. Two 50 m transects were surveyed at most sites (with a mean of 1.9 transects across all sites), predominantly on coral reef habitat, and generally parallel at different depths. Depth contours were restricted by depth variations in individual reefs, but where possible were selected to encompass a wide depth range (1–25 m). Constraints associated with diving bottom time and air consumption generally limited depths to less than 25 m. Photo-quadrats of the substrate were taken along each transect to quantify coral cover. These were taken vertically downward from a height sufficient to encompass an area of approximately 0.3 × 0.3 m, distributed every 2.5 m along each transect and later scored using a grid overlay of 5 points per image, totalling 100 points per transect.

Corals were identified to the lowest taxonomic resolution possible (generally species), leading to a total of 404 coral taxa. Given the high number of coral taxa (greater than recommended in most community ecology analyses) and their variable taxonomic resolution, these taxa were further grouped into a total of 119 coral categories by a single expert (E.T.) based on their genus/growth form combination. Note that if a genus only contains one species, the genus defines the coral category. Occasionally (<1% records), the genus could not be identified and was labelled as ‘coral’. The complete list of coral taxa is presented in electronic supplementary material, table S1. From the photo-quadrats, hard coral cover (%) was calculated for each coral category on each transect, as well as the sum of all coral categories (i.e. total live hard coral cover; %) after excluding non-coral categories (i.e. with percentage cover of all coral and non-coral categories summing up to 100% at each transect).

The severity of the 2016 marine heatwave at each site was estimated through two metrics of thermal stress: annual maximum DHW (°C-week) and annual maximum sea surface temperature anomaly (SSTA) observed in 2016. DHW represents the accumulation of positive temperature anomalies above the maximum monthly mean (MMM) SST + 1°C over a 12 week rolling window and is the most common predictor of coral bleaching risk [13]. Values of DHW >4°C-week typically correspond to a level 1 bleaching alert, and DHW >8°C-week correspond to a level 2 alert. By definition, however, DHW does not distinguish mild but prolonged heat stress from short but acute stresses, which can induce very different responses of corals in terms of bleaching and subsequent mortality (e.g. [33]). Therefore, we also included SSTA to capture this distinction. We extracted the maximum annual DHW and SSTA at each survey site for the 2016 marine heatwave from the National Oceanographic and Atmospheric Administration Coral Reef Watch v3.1 daily global 5 km satellite composite product [34] (electronic supplementary material, figure S1). DHW and SSTA were positively correlated across all survey sites (Pearson’s *r* = 0.15, *p* = 0.018). In addition to DHW and SSTA that reflected thermal stress experienced in 2016, we extracted the long-term mean annual sea surface temperature and its intra-annual (i.e. seasonal) s.d., as well as the MMM (i.e. with DHW accumulating above MMM + 1°C) from the NOAA Coral Reef Watch climatology (1985–2012).

Additional environmental covariates were obtained from the Marine Socio-Environmental Covariates dataset for the global oceans, which consists of environmental and anthropogenic variables summarized in ecologically relevant ways [35]. This dataset included four sets of environmental variables related to biophysical conditions (long-term mean and seasonal variation of net primary productivity corrected for shallow-water reflectance, wave energy including sheltered-coastline corrections) and landscape context (coral reef and land cover within a 20 km radius). All environmental covariates are described in table 1. Data used in this study are available online at http://doi.org/10.25909/23584173 [36].

**Table 1.** Environmental covariates used in multivariate regression trees (MRT) and boosted regression trees (BRT).

variable	description	unit	source	covariate in
SST_mean	sea surface temperature, climatological overall mean (1985–2012)	°C	NOAA Coral Reef Watch climatology	MRT
SST_sd	sea surface temperature, climatological intra-annual (seasonal) s.d. (1985–2012)	°C	NOAA Coral Reef Watch climatology	
NPP_mean	net primary productivity of carbon, overall mean (2003–2013)	mg C m^−2^ d^−1^	Yeager *et al*. [35]	
NPP_sd	net primary productivity of carbon, intra-annual (seasonal) s.d. (2003–2013)	mg C m^−2^ d^−1^	Yeager *et al*. [35]	
wave_mean	wave energy flux, overall mean	kW m^−1^	Yeager *et al*. [35]	
wave_sd	wave energy flux, intra-annual s.d.	kW m^−1^	Yeager *et al*. [35]	
reef_area	coral reef area within a 20 km radius	km^2^	Yeager *et al*. [35]	
land_area	land area within a 20 km radius	km^2^	Yeager *et al*. [35]	
depth	average transect depth at each site	m	this study	
region	categorical: GBR and Coral Sea/Ningaloo Reef/Northwest Shelf	—	this study	
DHW	degree heating weeks: maximum annual (2016)	°C-week	NOAA Coral Reef Watch	BRT
SSTA	sea surface temperature anomaly: maximum annual (2016)	°C	NOAA Coral Reef Watch	
SST_mmm	maximum monthly mean sea surface temperature (1985–2012)	°C	NOAA Coral Reef Watch	MRT + BRT

### (c) Statistical analyses

We first characterized pre-heatwave coral communities using multivariate regression trees (MRTs) [37], which model the relationship between ecological community composition and environmental covariates. MRT forms clusters of sites by repeated splitting of the data, with each split determined by environmental characteristics and corresponding to a distinct assemblage. We calibrated MRT using the pre-heatwave matrix of site-by-coral cover (square-root transformed) as the response variable and covariates describing long-term environmental conditions as predictors (table 1). We determined the best tree size (i.e. number of leaves or clusters formed by the tree) as that which minimized the cross‐validated relative error (CVRE), which varies from zero for a perfect predictor to nearly one for a poor predictor. We subsequently characterized each pre-heatwave coral community (i.e. cluster) by its indicator taxa based on the Dufrêne–Legendre index, which integrates the relative abundance and frequency of each coral category within a given cluster [38]. The index varies between 0, no occurrences of a coral category within a cluster, to 100, if a category occurs at all sites within the cluster and in no other cluster. The index is associated with the probability of output resulting from a random pattern, based on 250 reallocations of sites among clusters. MRT were fit in the R 4.0.2 [39] package ‘mvpart’, and the Dufrêne–Legendre index was calculated using the ‘labdsv’ R package.

Second, we quantified the change in coral community composition following the 2016 heatwave using distance-based redundancy analysis (db-RDA) [40], which is analogous to canonical analysis of principal coordinates (CAP) [41] in non-metric (distance-based) space. We used the Bray–Curtis dissimilarity matrix calculated from square root-transformed coral cover to reduce the influence of high values. For visualization purposes, the db-RDA ordination plots included total live coral cover, maxDHW and maxSSTA as illustrative covariates (i.e. these variables did not contribute to the ordination, but their position on the plot indicated how they correlated with changes in coral communities over time). We included a ‘pre/post heatwave’ factor associated with repeated surveys at each site to maximize the separation of sites in the biplot, as well as two constraints to control for the variable resampling interval between survey sites, and the time between the heatwave and the post-heatwave surveys, respectively. We tested whether the community composition of each cluster significantly changed between the pre- and post-heatwave surveys using a permutational multivariate analysis of variance (PERMANOVA) and the same constraints as in the db-RDA (here included as offsets). The PERMANOVA was conducted using the ‘adonis2’ function, the db-RDA was performed using the ‘capscale’ function, and illustrative covariates were added using the ‘envfit’ function, all in the R package ‘vegan’.

Using the same pre- versus post-heatwave coral categories-by-site matrices, we also calculated the temporal beta-diversity index (TBI) [42] using Bray–Curtis dissimilarity for each survey site as a proxy for the magnitude of change in assemblage composition between pre- and post-heatwave surveys. For each cluster, we tested the significance of change in coral cover (which underpins the calculation of TBI) using a permutational *t*‐test (*n* = 999 permutations). We verified the assumption that, for each survey site, TBI was correlated with the distance between ‘pre’ and ‘post’ heatwave site coordinates on the db-RDA ordination plot; the assumption of a strong correlation based on the fact that both variables are estimates of beta diversity [43]. We identified coral categories that significantly changed in percentage cover over time using a paired *t*‐test for each coral category corrected for multiple testing [42]. Being based on permutations (*n* = 999), this paired *t*‐test does not assume normality [42]. We re-ran the indicator taxa analysis (Dufrêne–Legendre index) on the post-heatwave community matrix to test for any changes in indicator taxa within each cluster following the heatwave. We also plotted the mean change in coral cover (%) for each coral category against the change in a number of sites at which that coral category occurred.

Lastly, we identified the main drivers of coral community response to disturbance among biotic (i.e. pre-disturbance community composition) and abiotic (i.e. thermal stress) variables using boosted regression trees (BRT). BRT are machine learning algorithms that use many simple decision trees to iteratively boost the predictive performance of the final models [44]. Model settings include the learning rate (lr) that controls the contribution of each tree to the final model and tree complexity (tc) that determines the extent to which interactions were fitted. The number of trees (nt) that achieved minimal predictive deviance (i.e. the loss in predictive performance because of a suboptimal model) was determined using cross-validation (function gbm.step with tc = 2, lr = 0.001 and bag fraction = 0.5) [44]. We ran two BRT models, using the absolute change in total hard coral cover (%) and the TBI, respectively, to test whether these response variables were explained by different predictors. BRT were fit in R using package ‘gbm’ and functions provided by Elith *et al*. [44].

## 3. Results

### (a) Structure of pre-heatwave communities

**Figure 1.**
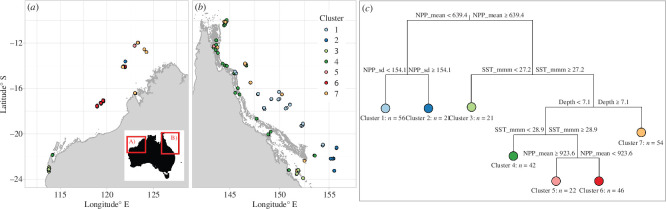
Spatial structure and environmental predictors of pre-heatwave coral communities identified by MRT. Clusters (1–7) are indicated for survey sites of the (*a*) northwest and (*b*) northeast Australian coasts. Major environmental predictors and values associated with each split of the trees are indicated in (*c*), with NPP_mean: net primary productivity; NPP_sd: intra-annual (i.e. seasonal) s.d. in net primary productivity; SST_mmm: maximum monthly mean sea surface temperature. See table 1 for details.

Pre-heatwave coral communities were strongly structured along latitudinal and longitudinal gradients at a regional scale, reflecting the major influence of environmental drivers including primary productivity and its seasonal variation, long-term MMM sea surface temperature and depth (figure 1).

A total of seven coral communities (‘clusters’) were identified using MRT across all survey sites, primarily split between low (clusters 1–2) and high (clusters 3–7) levels of net primary productivity. Central Coral Sea communities exposed to low, and seasonably stable, primary productivity (cluster 1) were not characterized by any indicator taxa prior to the heatwave (table 2). Indicator taxa of communities exposed to low, but seasonally variable primary productivity (cluster 2; mostly southern Coral Sea) included *Isopora* and *Porites* encrusting (i.e. *Porites lichen*; table 2). Among more productive environments, cooler and southernmost sites including Ningaloo Reef and the Capricorn Bunkers (cluster 3) were associated with greater representation of *Acropora* tabular and *Acropora* digitate (predominantly *Acropora digitifera*; table 2). Shallower sites of intermediate long-term sea surface temperature (cluster 4) represented mixed coral communities and were not characterized by any indicator taxa. The shallow, warmest sites were split between highly productive (cluster 5; Ashmore and Cartier) and less productive environments (cluster 6; Rowley Shoals and Scott Reef). Cluster 5 was characterized by the lowest hard coral cover with indicator taxa that included *Porites* branching (predominantly *Porites cylindrica*) and *Seriatopora*. Cluster 6 yielded the highest coral cover and included indicator taxa such as *Isopora* Branching (predominantly *Isopora brueggemanni*), *Porites* massive, *Acropora* staghorn and *Pavona* submassive (mostly *Pavona varians*). Finally, indicator taxa of deeper reefs (cluster 7) included *Montipora* encrusting, *Porites* submassive and *Pocillopora* branching (table 2). MRT explained a total of 13.9% variation in coral communities (CVRE).

**Table 2.** Indicator coral taxa associated with each coral community (cluster) and identified based on the Dufrêne–Legendre index (*p* < 0.05). Coral taxa that were an indicator pre- or post-heatwave are indicated as ‘Y’ in the ‘pre’ and ‘post’ columns, respectively. The term ‘predominantly’ is used when the taxon name covers a single species with a few exceptional records and ‘mostly’ when several other species are also important, as listed in order of occurrence. When neither ‘predominantly’ nor ‘mostly’ are specified, the listed species is the only indicator in that category.

clusters	indicator taxa	pre	post	main species
1	*Coscinaraea* submassive		Y	predominantly *C. columna*
2	*Isopora* columnar or digitate	Y	Y	*Isopora palifera*
	*Porites* encrusting	Y	Y	*Porites lichen*
	*Isopora* encrusting	Y	Y	mostly *I. palifera* and *I. cuneata*
	*Isopora* submassive	Y	Y	mostly *I. palifera* and *I. cuneata*
	*Acropora* arborescent table	Y		predominantly *A. abrotanoides*
	*Galaxea* branching^a^		Y	*Galaxea horrescens*
	*Millepora* submassive		Y	*Millepora exaesa*
	*Leptoria*		Y	*Leptoria phrygia*
3	*Acropora* digitate	Y	Y	predominantly *A. digitifera*
	*Acropora* tabular	Y	Y	*A. spicifera* in the west, other tables in the east
	*Echinopora* branching	Y	Y	*Echinopora horrida*
	*Montipora* foliose plate	Y	Y	mostly *M. aequituberculata*
	*Favites* hemispherical^a^	Y		
	*Platygyra* hemispherical	Y		predominantly *P. daedalea*
	*Galaxea* submassive	Y		predominantly *G. astreata*
	*Merulina* submassive		Y	predominantly *M. ampliata*
	*Acropora* corymbose		Y	mostly *A. tenuis*, *A. nasuta*, *A valida* and *A. seriata* in the West
	*Cyphastrea* submassive		Y	mostly *C. serailia*, *C. microphthalma* and *C. chalcidicum*
	*Lobophyllia* hemispherical		Y	*Lobophyllia hemprichii*
4	(none)			
5	*Ctenactis*	Y	Y	mainly *C. crassa* or *C. echinata*
	*Goniopora* hemispherical	Y	Y	mostly *G. columna*, *G. tenuidens*, *G. minor*, *G. lobata*
	*Porites* branching	Y	Y	predominantly *P. cylindrica*
	*Seriatopora*	Y	Y	*S. hystrix* and *S. caliendrum*
	*Oxypora* encrusting plate	Y		mostly *O. lacera*
	*Herpolitha*		Y	predominantly *H. limax*
	*Echinopora* plating		Y	*E. lamellosa* in the west and *E. pacificus* in the east
	*Goniopora* columnar or digitate		Y	generally unidentifiable to species
	*Platygyra* submassive		Y	*P. pini*, *P. lamellina*, *P. verweyi* and others
	*Mycedium* foliose plate		Y	*M. elephantotus* and *M. mancaoi*
6	*Isopora* branching	Y	Y	predominantly *I. brueggemanni*
	*Pavona* submassive	Y	Y	mostly *P. varians* and some *P. duerdeni*
	*Acropora* staghorn	Y	Y	mostly *A. muricata*, *A. abrolhosensis* and *A. intermedia*
	*Goniastrea* hemispherical	Y	Y	mostly *G. edwardsi*
	*Fungia*	Y		generally unidentifiable to species
	*Porites* massive	Y		one or more of the six massive forms^b^
	*Phymastrea* submassive	Y		predominantly *P. magnistellata*
	*Goniastrea* submassive		Y	*Goniastrea pectinata*
7	*Porites* submassive	Y	Y	*P. rus, P. vaughani* and *P. annae*
	*Pocillopora* branching	Y	Y	mostly *P. damicornis* and *P. verrucosa*
	*Montipora* encrusting	Y		generally unidentifiable to species
	*Heliopora*	Y		*Heliopora coerulea*
	*Physogyra*	Y		*Physogyra lichstensteini*
	*Porites* massive		Y	most likely the group of six—mostly *P. lutea*, *P. lobata*, *P. solida*, etc.
	*Leptastrea* encrusting		Y	most likely *L. purpurea*, *L. transversa* and *L. pruinosa*
	*Diploastrea*		Y	*Diploastrea heliopora*

^a^ Rare coral taxa that only occurred on a few transects in the corresponding cluster and nowhere else.

^b^ One or more of the six *Porites* species of massive growth form*—P. lobata, P. lutea, P. solida, P. australiensis, P. mayeri* and *P. myrmidonensis* [45].

### (b) Pre-heatwave versus post-heatwave changes in coral communities

**Figure 2.**
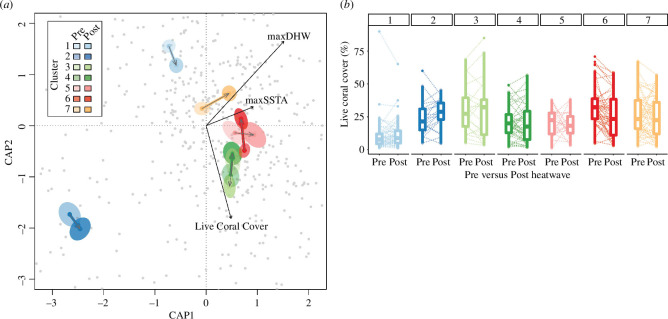
Change in coral communities before and after the 2016 marine heatwave. (*a*) db-RDA showing site ordination for both pre- and post-heatwave surveys (i.e. each site corresponds to two grey dots) and temporal change in coral community composition for each coral cluster (ellipses and arrows; see figure 1 for cluster description). Ellipses indicate the standard error across all survey sites within each cluster, and arrows link ellipses centroids for pre- and post-coral surveys. (*b*) Changes in percentage total live coral cover for sites within each cluster. Each site corresponds to one ‘pre’ and one ‘post’ observation linked by a dotted line. Boxplots show the median (horizontal line), interquartile range (hinges), 90% confidence interval (whiskers) and outliers (dots).

The 2016 marine heatwave had a highly variable footprint among different coral communities. Cluster 2 was the most distinct coral community, both pre- and post-heatwave, and experienced relatively little change in community composition (figure 2*a*) with a marginal increase in mean coral cover (*p* > 0.05; figure 2*b*). Clusters 1 and 3 also showed relatively little change in community composition (figure 2*a*) and were, like cluster 2, exposed to the lowest thermal stress (electronic supplementary material, figure S2). Some coral categories became indicators of clusters 2 and 3 only after the heatwave, including *Millepora* submassive (*Millepora exaesa*) and *Cyphastrea* submassive, respectively (table 2), despite both coral categories significantly decreasing over time across all sites (table 3).

**Table 3.** Coral taxa showing a significant change in percentage cover after the heatwave (*p* < 0.05; paired *t*‐test corrected for multiple testing). ‘Mean change’ indicates the mean percentage cover change across all sites, whereas ‘min change’ and ‘max change’ indicate the minimum and maximum percentage cover change at any one site, respectively. Changes in percentage cover for all coral categories are indicated in electronic supplementary material, table S2.

coral category	mean change	min change	max change	*t*.stat	*p*.perm
*Montipora* encrusting	−0.45	−10.6	7.0	−3.33	0.01
*Isopora* branching	−0.24	−21.2	6.8	−2.20	0.02
*Cyphastrea* submassive	−0.19	−4.0	1.0	−4.21	0.01
*Isopora* columnar or digitate	−0.18	−23.0	17.3	−2.16	0.01
*Millepora* submassive	−0.16	−13.7	5.0	−1.93	0.01
*Stylophora*	−0.14	−4.5	3.0	−2.45	0.01
*Porites* encrusting	−0.13	−11.0	5.5	−2.21	0.01
*Favites* submassive	−0.06	−3.0	3.5	−1.83	0.02
*Leptastrea* submassive	−0.05	−2.5	1.5	−1.54	0.03
*Coeloseris*	−0.04	−2.5	0.5	−3.41	0.01
*Echinopora* tubular	−0.04	−2.5	1.0	−2.02	0.03
*Astrea*	−0.03	−1.1	1.0	−2.07	0.01
*Tubipora*	0.02	−0.5	1.6	1.92	0.03
*Goniopora* columnar or digitate	0.03	−0.5	3.4	2.29	0.02
*Porites* massive	0.27	−19.0	21.0	1.72	0.02

By contrast, clusters 5–7 showed relatively higher coral community change (based on TBI and/or the pre/post distance on the redundancy analysis) after being exposed to greater levels of heat stress (figure 2*a*; electronic supplementary material, figure S2). However, such community composition change over time was only significant for cluster 7 based on both the PERMANOVA (*p* = 0.001) and the permutational *t*‐test (*p* = 0.01). Coral cover marginally declined within all three clusters, albeit only significantly for cluster 6 (*p* < 0.05; figure 2*b*). Interestingly, *Porites* massive was an indicator for cluster 6 prior to the heatwave, but for cluster 7 after the heatwave (table 2).

We found strong relationships between the two metrics of beta diversity, showing consistent responses to the heatwave. Specifically, the TBI was strongly related to the distance between pre- and post-heatwave site coordinates on the db-RDA ordination plan (Pearson’s *r* = 0.38, *p* < 0.001; electronic supplementary material, figure S3*a*). TBI was also correlated with the absolute change in total hard coral cover, albeit to a lesser extent (*r* = 0.17; *p* = 0.006; electronic supplementary material, figure S3*b*), suggesting that changes in coral community composition were partly decoupled from the magnitude of coral cover change (whether corresponding to an increase or decline) observed at the survey sites.

### (c) Drivers of coral community responses to the heatwave

**Figure 3.**
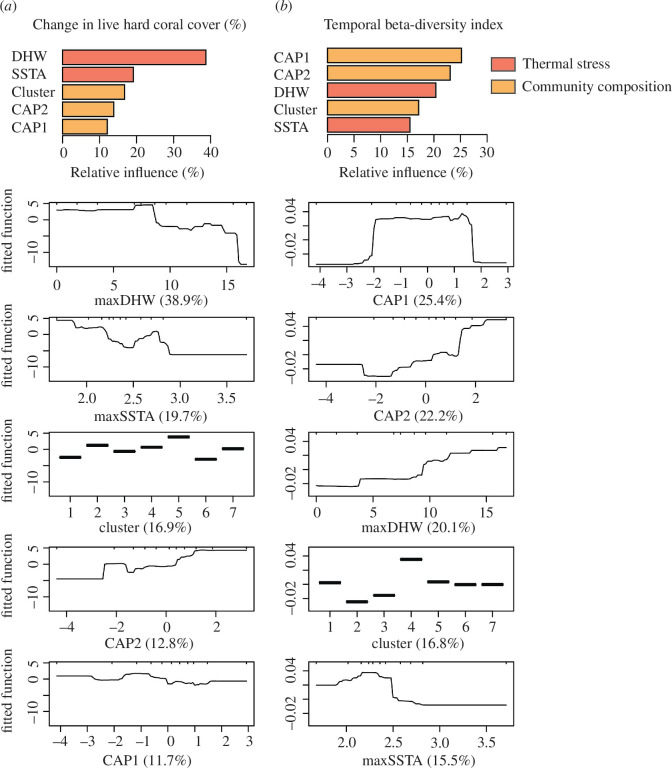
Influence of thermal stress and pre-heatwave community composition on coral responses to the 2016 marine heatwave based on (*a*) the change in live hard coral cover and (*b*) the TBI. The bar plot (top panel) shows the relative importance of different predictors (%), and the bottom panels show marginal responses to each predictor, as determined by BRT. Thermal stress metrics include maximum DHW and SSTA. Proxies for pre-heatwave community composition included the coral cluster (figure 1) as well as the pre-heatwave site coordinates on the db-RDA site plot (CAP1 and CAP2; figure 2).

BRT identified very different drivers of the change in live hard coral cover and the TBI (figure 3). Thermal stress variables were the primary drivers of total coral cover change, with DHW and SSTA totalling a relative importance of 58.6%, and step declines in coral cover at DHW > 8 and 16°C weeks (noting the latter was only due to 10 sites), and SSTA > 2.2°C. A total of 44.2% deviance in total coral cover change was explained by BRT. In contrast, proxies for the pre-heatwave community composition were the best predictors of the TBI (combined relative importance = 64.4%), with a total of 39.1% deviance explained by all predictors collectively. TBI was the highest at CAP1 scores ranging from 0 to 2 (mostly corresponding to clusters 6 and 7, with 29 and 19% of all sites within this range, respectively) and higher scores on CAP2 (mostly clusters 1 and 7, with 63 and 18% of all sites with CAP2 > 1, respectively). DHW was the third most important predictor of coral community composition, with a positive effect on TBI (i.e. greater change in community composition at higher levels of heat stress), followed by the cluster (i.e. greater change in community composition for cluster 4) and SSTA (figure 3).

### (d) Winners and losers

**Figure 4.**
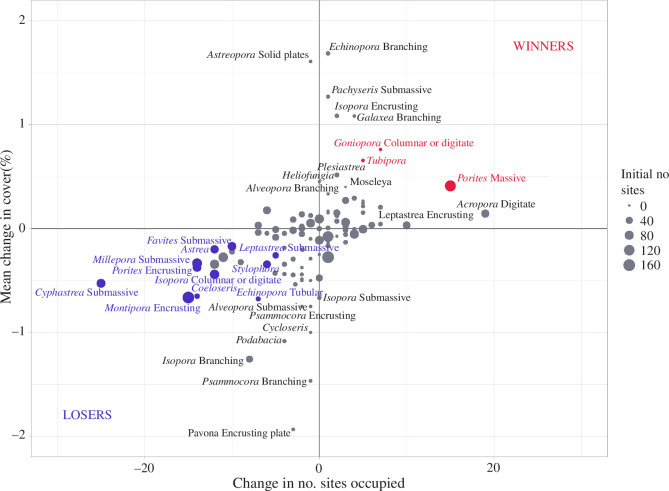
Winners (red) and losers (blue) of the 2016 marine heatwave. Dots indicate coral categories ordinated based on the change in number of sites occupied (*x*-axis) and the mean change in percentage cover across all sites (*y*-axis). Coral categories are colour-coded only where changes in percentage cover over time are significant and unlabelled in the central region of the plot for clarity. Dot size is proportional to initial number of sites occupied.

Most coral categories that decreased in percentage cover also decreased in number of sites at which they were recorded, and vice versa (figure 4). Coral categories with the most pronounced changes (either increasing or decreasing) were also those with significant changes in percentage cover after the heatwave, including three coral taxa ‘winners’ (*Porites* massive, *Tubipora*, *Goniopora* columnar or digitate) and 12 coral taxa ‘losers’ (table 3). *Acropora* staghorn and *Acropora* tabular showed the largest decline and largest increase at any one site (−36 and +34%, respectively, both on the Great Barrier Reef; electronic supplementary material, table S2) but showed no significant changes once averaged across all sites. Pre- and post-heatwave mean coral cover in each cluster, as well as their mean difference, are presented for all taxa in electronic supplementary material, table S2. It should be noted that low values of mean coral cover change in table 3 and electronic supplementary material, table S2 are due to (i) these mean changes being calculated across all sites, including some where the coral category does not occur, and (ii) the relatively high number of detailed coral categories considered here (*n* = 119) that, once considered alongside non-coral benthic categories to calculate percentage covers, result in low numbers overall.

## 4. Discussion

Understanding how coral responses to marine heatwaves vary among taxa is essential if we are to anticipate future community composition and potential ecological consequences as ocean warming intensifies (e.g. [1,46]). Using a continental-scale, high taxonomic resolution dataset of coral covers before and after the major 2016 marine heatwave on tropical Australian reefs, we show that the pre-heatwave community composition, which was strongly structured along environmental gradients, was a better predictor than the magnitude of heat stress in explaining how coral communities were impacted by disturbance. Furthermore, we identified different indicator coral taxa before and after the heatwave in each community, therefore detecting potential winners and losers of heat stress and an indication of how communities are likely to reorganize as marine heatwaves intensify in frequency and severity.

Our results showed that the pre-heatwave community composition largely dictated how communities subsequently changed following the heatwave, whereas the magnitude of thermal stress was the primary driver of total coral cover change. Pre-heatwave community composition reflects a combination of spatial gradients in environmental conditions and historical exposure to disturbance [11,47,48]. Such exposure to disturbance can affect community composition through environmental filtering [16,49], whereby previous disturbances have reduced the abundance of the most sensitive taxa, leaving more robust ones that are less likely to be impacted by subsequent disturbances [50]. Indeed, coral life history strategies (e.g. competitive, weedy and stress-tolerant [19]) can help understand and predict how different coral taxa respond to heat stress, although these strategies remain to be quantified for most coral taxa of the world (but see e.g. [20]). Heat tolerance in reef-building corals is also notoriously complex as it not only varies among but also within species (e.g. due to long-term genetic adaptation, variation in symbiont community structure and/or colony size and life stage) and between regions depending on their exposure to thermal stress [12,15,21,51,52]. In our study, DHW proved to be an effective predictor of such exposure to thermal stress, with BRT showing a sharp decline in coral cover after 8 and 16°C-weeks, and an almost linear pattern of change in community composition. These results support the ecological relevance of DHW across large geographical scales [53].

We also found that coral community composition was largely structured along environmental gradients. This was primarily associated with the long-term mean primary productivity and its seasonal variability, as well as the climatological MMM sea surface temperature, which defines the community-specific bleaching threshold [13]. These environmental gradients can result in sharp latitudinal and cross-shelf distinctions between coral communities (e.g. [54,55]). Similar distinctions have been inferred on the Great Barrier Reef based on other environmental predictors not directly accounted for here (e.g. water quality [24,56]). Interestingly, the importance of seasonal variation in net primary productivity observed in offshore reefs of the Southern Coral Sea (cluster 2) was consistent with the occurrence of seasonal upwellings [57]. These reefs were also exposed to relatively low heat stress during the 2016 heatwave and showed a marginal increase in hard coral cover associated with a low temporal turnover in community composition after 2016. Surprisingly, a depth of >7 m (cluster 7) did not seem to confer a protective buffer against heat stress since temporal turnover was significant in this cluster only. While reduced light in deeper environments can moderate coral mortality during heat stress, which has been documented both in physiological [58] and molecular studies [59], recent severe bleaching events were found to impact deeper corals as well [60]. These results point to the importance of understanding marine microclimates, where the degree of natural variation in environmental conditions can either pre-adapt ecological communities to thermal stress (i.e. upwellings) or protect them from it (i.e. deeper environments) [61]. Such marine microclimates can provide spatial refuges or safe havens even under extreme events (e.g. El Niño), although their persistence under future climate change scenarios remains to be tested beyond regional scales [62,63].

Our analysis of indicator taxa in each coral community showed differing responses to the 2016 heatwave; 11 coral taxa were indicator before the heatwave but not after, 17 taxa became indicators only after the heatwave and 18 did not change status. Taxa that decreased in cover or were no longer indicators included those with complex morphologies such as *Acropora* tabular and *Acropora* arborescent table, or *Isopora* branching. Similarly, coral bleaching impacts in the Coral Sea tended to be lower at sites dominated by *Porites* and higher at those dominated by *Stylophora*, *Pocillopora* and *Acropora* [64], which was also observed in the Western Indian Ocean [65]. Overall, these results corroborate the idea that the 2016 marine heatwave might have induced a shift away from communities dominated by fast-growing, branching and tabular coral taxa that provide important habitat for reef-associated organisms, to assemblages of taxa with massive growth forms and slower growth rates [22]. This general community response was nevertheless not ubiquitous, as some *Acropora* taxa remained indicators after the heatwave (table 2), and not all *Acropora* categories declined consistently (electronic supplementary material, table S2). In addition to concurrent, yet patchy declines in coral cover, these shifts in coral communities and associated growth forms might have long-lasting impacts on associated reef organisms, including fish communities that have already shown signs of ecological generalization in response to the 2016 heatwave and associated mass coral bleaching event [4,66].

Several potential caveats should be considered when interpreting our results. First, although unlikely, other undetected disturbances between the pre- and post-heatwave surveys could have clouded our interpretation of the impact of the 2016 heatwave. It is also possible that a few reefs on the Northwest Shelf, impacted by Tropical Cyclone Narelle a few months prior to the 2013 pre-heatwave surveys, were only just starting to recover from cyclone impacts; however, the low number of these reefs should not have had a drastic influence on our results. Given that the 2016 heatwave was subsequently followed by a series of other heatwaves [16,22,60], it will be important to study their respective impacts in a cumulative disturbance framework. Second, stochastic demographic processes (such as natural coral mortality or resulting from, e.g. disease) could have affected the change in coral cover over this period, although it can be assumed that this effect would be evenly distributed across the dataset (i.e. with no bias in any particular direction). A similar assumption can be made about possible measurement error, whereby a slight variation in the location of transects and photo-quadrats between surveys could explain occasionally large (approx. 20%) increases in the cover of a given category at a single site. Finally, while our approach of categorizing coral assemblages by clusters was useful in attempting to simplify a complex picture of variation in both assemblage composition and exposure to heat stress, we recognize that the transferability of our results and conclusions might be limited by the idiosyncrasies of our study region, timing of the surveys and corresponding coral assemblages. Indeed, the composition of pre-disturbance communities was likely influenced by the successional state of coral communities following past disturbances, for which data were not consistently available throughout the entire study area. For example, previous studies showed that, prior to 2008, reefs in the mid and offshore central-north GBR were dominated by tabular *Acropora* when coral cover exceeded 30% [67]. The different patterns we observed here are likely influenced by ecological successions within coral communities following disturbances that have occurred since 2008.

Our assessment of taxon-specific responses to a major heatwave suggests several promising new pathways for future research. Firstly, assessment of such responses could feed into the development of a species-level indicator of coral vulnerability to thermal stress, pending the sufficient availability of similar datasets at the species level. Novel Bayesian joint species distribution modelling frameworks can now account for species interactions while quantifying species responses to environmental gradients [68] and allow the incorporation of relevant species traits such as bathymetric or thermal range of occurrence [20]. However, the development of robust estimates of species-level vulnerability to thermal stress will rely upon a longer time series of ecological data that span multiple disturbance events, including tropical cyclones [31], or outbreaks of the CoTS *Acanthaster* spp. [69,70] or coral disease [71], which were beyond the scope of this study. Longer ecological time series will also be essential to understand how communities reorganize over the years following a disturbance [72]. The successful development of species-level (or genus-level) indicators of coral vulnerability to thermal stress could open important avenues for future coral conservation, for example, to prioritize small-scale coral restoration efforts or genetic manipulation [73–75], as well as providing a better understanding of the mechanisms that underpin higher resistance to heat stress (e.g. short-term acclimation, long-term genetic adaptation and/or variation in symbiont community structure) [76,77].

In conclusion, coral community reshuffling in response to the 2016 heatwave was largely decoupled from changes in total coral cover and likely driven by a complex interplay between species life history, local reef environment, disturbance history and the stage of coral recovery. As marine heatwaves intensify in frequency and severity, an important research priority will be to derive transferrable indices of taxon-specific coral vulnerability, coupled with future forecasts of thermal stress and climate refugia, to guide future coral reef conservation and management.

## Data Availability

The data that support the findings of this study can be accessed through Figshare at [36]. The code used for the analysis is available at [78]. Supplementary material is available online [79].
